# Worldwide impact of lifestyle predictors of dementia prevalence: An eXplainable Artificial Intelligence analysis

**DOI:** 10.3389/fdata.2022.1027783

**Published:** 2022-12-08

**Authors:** Loredana Bellantuono, Alfonso Monaco, Nicola Amoroso, Antonio Lacalamita, Ester Pantaleo, Sabina Tangaro, Roberto Bellotti

**Affiliations:** ^1^Dipartimento di Biomedicina Traslazionale e Neuroscienze (DiBraiN), Università degli Studi di Bari Aldo Moro, Bari, Italy; ^2^Istituto Nazionale di Fisica Nucleare, Sezione di Bari, Bari, Italy; ^3^Dipartimento Interateneo di Fisica, Università degli Studi di Bari Aldo Moro, Bari, Italy; ^4^Dipartimento di Farmacia-Scienze del Farmaco, Università degli Studi di Bari Aldo Moro, Bari, Italy; ^5^Dipartimento di Scienze del Suolo, della Pianta e degli Alimenti, Università degli Studi di Bari Aldo Moro, Bari, Italy

**Keywords:** dementia, eXplainable Artificial Intelligence, complex systems, One Health, sustainable development goals, data science for social good, computational social science, AI for social good

## Abstract

**Introduction:**

Dementia is an umbrella term indicating a group of diseases that affect the cognitive sphere. Dementia is not a mere individual health issue, since its interference with the ability to carry out daily activities entails a series of collateral problems, comprising exclusion of patients from civil rights and welfare, unpaid caregiving work, mostly performed by women, and an additional burden on the public healthcare systems. Thus, gender and wealth inequalities (both among individuals and among countries) tend to amplify the social impact of such a disease. Since at present there is no cure for dementia but only drug treatments to slow down its progress and mitigate the symptoms, it is essential to work on prevention and early diagnosis, identifying the risk factors that increase the probability of its onset. The complex and multifactorial etiology of dementia, resulting from an interplay between genetics and environmental factors, can benefit from a multidisciplinary approach that follows the “One Health” guidelines of the World Health Organization.

**Methods:**

In this work, we apply methods of Artificial Intelligence and complex systems physics to investigate the possibility to predict dementia prevalence throughout world countries from a set of variables concerning individual health, food consumption, substance use and abuse, healthcare system efficiency. The analysis uses publicly available indicator values at a country level, referred to a time window of 26 years.

**Results:**

Employing methods based on eXplainable Artificial Intelligence (XAI) and complex networks, we identify a group of lifestyle factors, mostly concerning nutrition, that contribute the most to dementia incidence prediction.

**Discussion:**

The proposed approach provides a methodological basis to develop quantitative tools for action patterns against such a disease, which involves issues deeply related with sustainable, such as good health and resposible food consumption.

## 1. Introduction

The term *dementia* indicates a group of diseases that progress over time and affect cognitive abilities, memory, and behavior, ultimately interfering with the capability to carry out daily activities (Reitz et al., 2011; Arvanitakis et al., 2019). Dementia cases worldwide are constantly increasing and it is estimated that by 2050 they will exceed 139 millions (WHO, 2022a). Dementias show a greater impact on the world population than other diseases that affect the central nervous system, such as brain cancer, for which about 300,000 cases were diagnosed in 2020 (IARC, 2020) and an annual growth rate of 1.50% is expected in the U.S. until 2030 (GlobalData, 2021). Alzheimer's disease (AD) is the most common form of dementia with over 70% of cases (Zhong et al., 2015; Pistollato et al., 2016; Silva et al., 2019), followed by vascular dementia (15–30%) (Wolters and Ikram, 2019), dementia with Lewy bodies (3.2–7.1%) (Hogan et al., 2016a) and frontotemporal dementia (0.01–4.6%) (Hogan et al., 2016b), and generally it has mild initial symptoms, such as memory alterations, that worsen incrementally. The progression of the disease involves behavioral changes and, in the final stages, the patient becomes completely dependent. Currently, there is no cure for AD but rather drug treatments to mitigate symptoms, with a limited therapeutic and temporal range (Mosconi and McHugh, 2015; Porsteinsson et al., 2021). Overall dementia-related costs in 2015 were estimated at 818 billion USD, namely 1.1% of global GDP, including direct medical costs, social care costs, and caregiver income losses (WHO, 2015; El-Hayek et al., 2019). The proportion of such costs is widely varying according to the country development status: in high-income states, the dementia-related costs are essentially divided between informal care (45%) and social care (40%); in low- and middle-income countries, instead, social care costs (15%) are much smaller than informal care costs (Prince et al., 2015b). Such a discrepancy contributes to increase inequalities among different areas and populations, especially considering that nearly 60% of people with dementia currently lives in low- and middle- income states (Prince et al., 2015a).

Besides the individual health issues and the social costs, dementia entails a series of collateral problems, prominently including the fact that unpaid care is often a burden of family women, which exacerbates the gender gap in the labor markets (Anderson and Oderkirk, 2015), as well as the exclusion from basic social and human rights of the affected people (WHO, 2017a), especially in contexts where they are passive and dependent recipients of care (Young et al., 2019). Therefore, increasing knowledge on dementia and finding new solutions to tackle the problem must be considered a priority also in view of the Sustainable Development Goals (SDGs) defined in the United Nations 2030 Agenda (United Nations Department of Economic and Social Affairs, 2021). Actually, for the reasons outlined above, the issue of dementia is connected not only to SDG 3 “Good Health and Well-Being,” which has the purpose to “ensure healthy lives and promote well-being for all at all ages,” but also to SDG 5 “Gender equality,” and to SDG 10 “Reduced Inequalities,” that specifically refers to the need of empowering inclusion of people with disabilities at all levels.

Although there are no certainties about the origin of the most relevant forms of dementia, including AD, several genetic and environmental factors that could influence its appearance and evolution are being examined (Fern and Ruiz-Gabarre, 2019; Bello-Corral et al., 2021). Despite age being the strongest risk factor, it is increasingly clear that dementia is not an inevitable consequence of biological aging, and can have a juvenile onset with symptoms appearing before the age of 65. Today the focus is on a number of additional lifestyle risk factors that include smoking (Batty et al., 2014; Zhong et al., 2015), alcohol abuse, unbalanced diets (Gardener and Rainey-Smith, 2018; Moore et al., 2018; van den Brink et al., 2019; Zhang et al., 2021), obesity (Singh-Manoux et al., 2018; Ma et al., 2020), physical inactivity, high blood sugar or cholesterol values, mid-life hypertension. Furthermore, low educational attainment, cognitive inactivity, social isolation and mid-life depression, as well as environmental conditions such as air pollution, are considered as potentially modifiable risk factors specific to dementia (WHO, 2022a).

The complex and multifactorial etiology of dementia, resulting from an interplay between genes and the environment, makes the study in this field particularly suited to the multidisciplinary “One Health” approach, aimed at designing and implementing programmes, policies, legislation and research in which multiple sectors, ranging from basic sciences and clinical studies to health-services policy analyses, cooperate together to achieve better public health outcomes (WHO, 2017b). Specifically, modern health research on dementia can benefit from the Big Data framework, due to the combined availability of *broad* and *deep data*: the former consist in massive amounts of routinely-collected population-based outcome and exposure data, while the latter provide clinical and biological information on individuals (Deetjen et al., 2015).

In the present research, we focus on broad data, applying methods of Artificial Intelligence (AI) and complex systems physics to predict the incidence of dementia from a set of variables, concerning individual health, food consumption, substance use and abuse, efficiency of the healthcare system. The analysis will be made at the level of countries, using publicly available indicators referred to a time window of 26 years, collected either directly from the Global Health Observatory data repository of the World Health Organization (WHO, 2022b) or from the Our World in Data repository (Global Change Data Lab, 2022), which includes data published by different international organizations.

Among the considered features, we will investigate the most influential ones in determining dementia prevalence in the population of each country, at different years. Specifically, we shall predict the dementia incidence through a Random Forest algorithm (Breiman, 2001), and evaluate the impact of the different lifestyle indicators on each prediction by means of eXplainable Artificial Intelligence (XAI) (Adadi and Berrada, 2018), an innovative approach that allows to improve the interpretability and transparency of Machine Learning models.

To increase the robustness of our analysis, we will employ the tool of complex networks (Newman, 2018), that is increasingly used, also in its recent formal developments (Bianconi, 2018; Amoroso et al., 2020, 2021b), along with or in replacement of traditional statistical techniques to unveil hidden information in many sectors of science and society, such as neuroscience (Sporns, 2011; Amoroso et al., 2018, 2019; Bellantuono et al., 2021), genetics (Monaco et al., 2019, 2020), economics (Hidalgo et al., 2007; Battiston et al., 2012; Tacchella et al., 2012; Hausmann et al., 2014; Bardoscia et al., 2017, 2021; Pugliese et al., 2019; Amoroso et al., 2021a), human mobility (Alessandretti et al., 2018), social and development-related issues (Bellantuono et al., 2020, 2022a,b).

The adoption of a framework combining XAI with complex system methods will lead us to first identifying predictors of dementia prevalence, and then verifying their occurrence in literature as acknowledged or suspected risk factors. The proposed workflow thus provides a possible methodological basis to develop quantitative tools for action patterns against such a problem, that involves issues deeply related with sustainable development, such as good health and, as we shall see, also responsible food consumption.

The article is organized as follows. In Section 2, we present details on data collection and processing, and on the machine learning, complex networks and XAI machinery employed in our research. The main results of this work are reported in Section 3. Finally, we discuss our findings in Section 4, pointing out their relation with previous literature and the insights they provide in terms of dementia prevention and its relation with sustainable development.

## 2. Materials and methods

**Figure 1:**
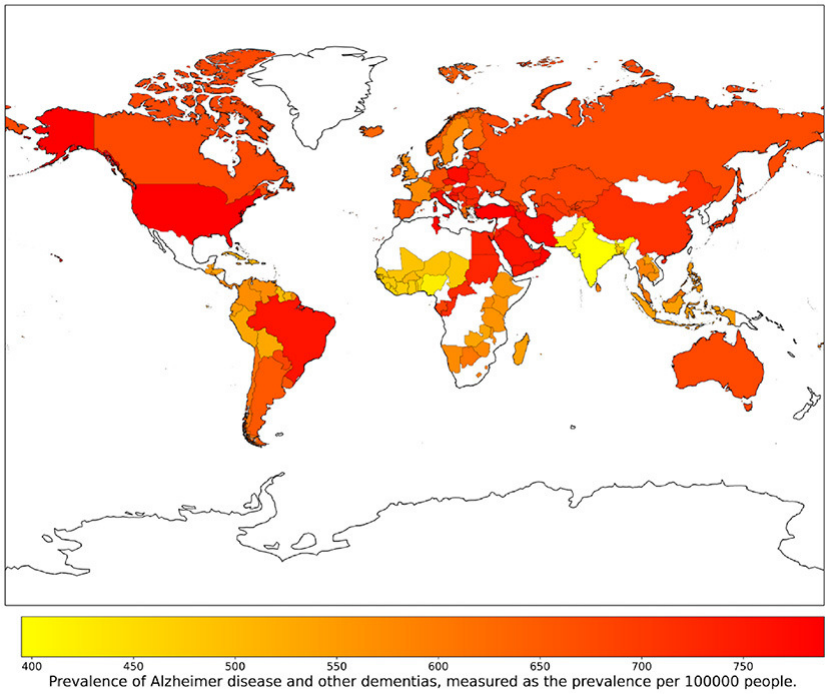
Prevalence of AD and other forms of dementia (PAD) in 137 UN countries for the year 2003.

**Figure 2:**
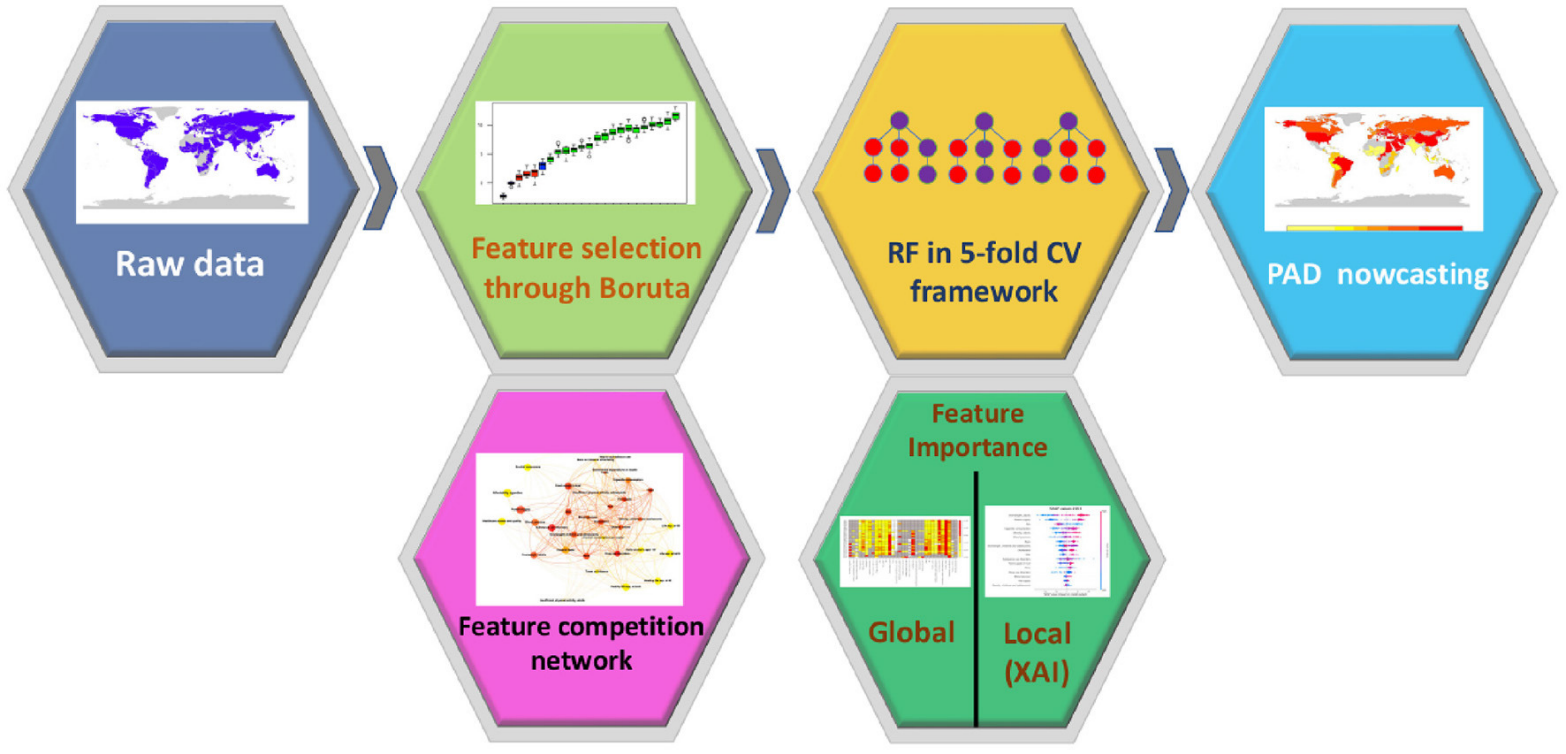
Flowchart of the proposed analysis. After a pre-processing phase, we applied a machine learning framework to predict the PAD for 137 UN countries between 1993 and 2019. In addition, we implemented a feature selection procedure to assess the role of each feature in the model.

The goal of our study was to explore whether social, economic, clinical and lifestyle factors could predict the Prevalence of AD and other forms of dementia (that we indicate as PAD) in 137 UN countries from 1993 to 2019. The choice of focusing on data referred to different geographical contexts and years is aimed at obtaining a broad and varied perspective. PAD is measured as the prevalence per 100, 000 people and age-standardized, thus allowing for a fair comparison between countries and through time. Values of this index for the considered countries and the year 2003 are represented in Figure 1. This map can be regarded as representative of the whole period 1993–2019, since, in this time span, variations of PAD throughout the years are very limited: the median standard-deviation-to-mean ratio is 1.10%, with the largest value occurring for Japan (6.59%), and the smallest for Iceland (0.15%). For the prediction of the PAD, we use a machine learning approach, as summarized in Figure 2. After a preprocessing phase, we select the most informative features through the wrapper method Boruta. Based on Boruta's output, we build a feature competition network and feed a machine learning framework based on the Random Forest algorithm. Finally, we implement a feature importance procedure using two different approaches: i) global, with Random Forest internal functionalities; ii) local, based on the Shapley (SHAP) values method.

### 2.1. Data collection and preprocessing

In this study, we consider 34 intensive indicators (i.e., not proportional to either the population or the extension of a country) referred to the 137 United Nations (UN) Member States highlighted in Figure 1. We collect data for the years 1993–2019 from two public repositories, namely the Global Health Observatory data repository of the World Health Organization (WHO, 2022b) and the Our World in Data repository (Global Change Data Lab, 2022), which includes data published by different international organizations.

Both indicators and countries are included in this study considering data availability, which can fluctuate from 1 year to another. In the Supplementary material, we report all the indicators that we use to predict the PAD, together with information on the related sources and availability throughout the years. Since the dataset refers to a time span of 26 years, in which certain political borders or denominations have changed, we operate on input data to align country names to their current version (see Supplementary material).

In the dataset of 34 indicators for 137 countries, missing data occur throughout the availability period, amounting to about 2% of entries. We follow a preprocessing strategy to fill these gaps, summarized in these steps:

- For each pair (indicator and country) with at least 5 values in the availability period, and without any consecutive missing entries at its ends, we interpolate the indicator using a quadratic fit model with the year as independent variable.
- For other (indicator,country) pairs, missing values are replaced with the average of the available ones.

At the end of the aforementioned filling operation, we standardize over all states the distribution *F*_*i,y*_ = {*f*_*i,s*_(*y*)}_1≤*s*≤137_ of the values of feature *f*_*i*_ in the year *y*, by redefining entries as

(1) $$\begin{array}{l} {\tilde{f}}_{i,s}\left(y\right)=\frac{f_{i,s}\left(y\right)-mean\left(F_{i,y}\right)}{\sqrt{var\left(F_{i,y}\right)}}. \end{array}$$

The standardized feature vector is used as input of the Boruta algorithm, that performs feature selection.

### 2.2. Feature selection

We implemented a feature selection wrapper method based on the Boruta framework (Kursa and Rudnicki, 2010) to reduce noise and redundant data, selecting only the uncorrelated features that improve the performance of a machine learning algorithm. Boruta is a robust and efficient feature selection tool that relies on a supervised learning Random Forest (RF) algorithm. In particular, Boruta exploits the founding concept of RF, according to which the negative effect of random fluctuations and correlation inherent in the learning model is mitigated by randomly perturbing the system and randomizing the training samples.

The method is based on attaching to the original set of features an equal number of *shadow* features, obtained by randomly shuffling the values of each original indicator. The extended dataset is used to feed a RF algorithm, which predicts a given quantity and evaluates the importance of both original and shadow features. After a series of independent random shuffling operations, Boruta selects the features that are more important, in a statistically significant way, than their respective shadow counterparts. In this way, such a feature selection method overcomes the limitations of classical techniques, where, since features compete with each other, an arbitrary importance threshold must be set to select relevant variables.

### 2.3. Feature competition network based on Boruta importance rankings

By comparing the Boruta feature importance rankings for different years, we construct a competition network (Criado et al., 2013; Fernández Tuesta et al., 2020), in which nodes represent features, and the strength of their connection is related with the tendency to switch their positions in the importance rankings associated to different years and countries. In such a picture, outstandingly relevant features, which tend to stay on top of the rankings for most years, are characterized by weak network connections. Competition networks provide a convenient tool to visualize and interpret the results of feature importance analysis, highlighting the competition patterns among different indicators and characterizing the stability of their hierarchies in the Boruta rankings across different years. Actually, encoding this information in a quantitative way is particularly useful when feature availability widely varies from 1 year to another. In this study, competition networks allow to combine information on availability and relevance of features in the rankings referred to a time span of 26 years. Features that are generally most relevant for prediction can be thus identified as the ones characterized at the same time by low network connectivity and high importance value.

For each pair of features *f*_*i*_(*y*) and *f*_*j*_(*y*) available to the Boruta algorithm for predicting the dementia incidence in a given year *y*, we consider the respective Boruta importances *I*(*f*_*i*_(*y*)) and *I*(*f*_*j*_(*y*)). These quantities provide the basis to construct the vector *V*(*f*_*i*_, *f*_*j*_), whose entries correspond to the set of years *Y*(*f*_*i*_, *f*_*j*_) in which both features *f*_*i*_(*y*) and *f*_*j*_(*y*) are available to the Boruta algorithm. Specifically, the entry *V*_*y*_(*f*_*i*_, *f*_*j*_) of *V*(*f*_*i*_, *f*_*j*_) corresponding to a given year *y* ∈ *Y*(*f*_*i*_, *f*_*j*_) reads

(2) $$\begin{array}{l} V_{y}\left(f_{i},f_{j}\right)=\{\begin{array}{cc} 1 & \text{if}I\left(f_{i}\left(y\right)\right)>I\left(f_{j}\left(y\right)\right)\\ 0 & \text{if}I\left(f_{i}\left(y\right)\right)\leq I\left(f_{j}\left(y\right)\right) \end{array} \end{array}$$

The variety of entries in *V*(*f*_*i*_, *f*_*j*_) is quantified by its Shannon entropy *E*(*f*_*i*_, *f*_*j*_). Hence, if the feature *f*_*i*_(*y*) has a higher Boruta importance than *f*_*j*_(*y*) in the prediction of dementia incidence for all years *y* ∈ *Y*(*f*_*i*_, *f*_*j*_), then all the entries of *V*(*f*_*i*_, *f*_*j*_) will be equal to zero, and *E*(*f*_*i*_, *f*_*j*_) = 0. The same occurs if the Boruta importance of *f*_*j*_(*y*) is always higher than the one of *f*_*i*_(*y*), with all the entries of *V*(*f*_*i*_, *f*_*j*_) being equal to 1.

The competition network consists of nodes, coinciding with the features *f*_*i*_ which are available to the Boruta algorithm at least for 1 year, and weighted edges (*f*_*i*_, *f*_*j*_, *w*_*ij*_), with weights *w*_*ij*_ = *E*(*f*_*i*_, *f*_*j*_). If *w*_*ij*_ = 0, there is no link between *f*_*i*_ and *f*_*j*_, i.e. these features do not compete with each other.

### 2.4. Learning model

Based on the evaluated importance, the Boruta algorithm selects a subset of relevant features for each year. We use selected features to feed a Random Forest model. Random Forest (RF) (Breiman, 2001) is one of the most used algorithms in supervised machine learning applications due to its versatility, ease of tuning, and ability to model multimodal data. A RF configuration depends on only two parameters: (i) the number of trees of the forest, *M*; (ii) the number of features to be chosen randomly at each split, *f*. RF comprises an ensemble of classification and regression trees (CART) that is grown in the training phase through a bootstrap process and a feature randomization procedure; this procedure makes RF robust against overfitting and only loosely correlated regressor trees. Another important advantage of RF is the possibility to internally evaluate the role of each feature for the model accuracy. In the present work we assess the feature importance using the mean decrease impurity and set a RF standard configuration with *M* = 500 trees and *f* = *F*/3, where *F* is the number of input features.

To further increase the robustness of our procedure we implement a 5-fold cross validation (CV) scheme repeated 100 times. The average of the 5 × 100 performance values obtained with this approach is a reliable indicator of the overall model performance. Similarly, RF feature importance is assessed through 100 CVs, and overall feature importance is computed by averaging. We measure performances in terms of coefficient of determination between predicted and actual values (*R*^2^). In addition, we evaluate the root mean square error (*RMSE*), defined as

(3) $$\begin{array}{l} RMSE=\sqrt{\frac{1}{n}\overset{n}{\underset{i=1}{\sum }}{\left(A_{i}-F_{i}\right)}^{2}}, \end{array}$$

and the mean absolute percentage error (MAPE), defined as (de Myttenaere et al., 2016):

(4) $$\begin{array}{l} MAPE=\frac{1}{n}\overset{N}{\underset{i=1}{\sum }}|\frac{A_{i}-F_{i}}{A_{i}}| \end{array}$$

where *A*_*i*_ is the actual value and *F*_*i*_ is the forecast value. Data processing and statistical analyses have been performed in R 3.6.1 (R Core Team, 2018) and Python 3.7.

### 2.5. eXplainable Artificial Intelligence and Shapley values

The XAI framework meets the crucial need to increase transparency and interpretability of Machine Learning models, especially relevant in their real-life applications (Miller, 2019; Bussmann et al., 2020; Jiménez-Luna et al., 2020; Lombardi et al., 2021). While, historically, a major role in developing AI models has been played by informativeness, quantified through performance metrics, and uncertainty estimation (Schaffer, 1993; Rao et al., 2008; Musil et al., 2019), an increasing attention is paid nowadays to the matters of generalization (reliability of predictions on previously unseen data) and transparency (ability to make the decision process as intelligible as possible) (Flach, 2019; Vollmer et al., 2020). XAI represents the common label of a framework of techniques that follow a unified view, which combines informativeness, uncertainty estimation, generalization, and transparency. In this work, we adopt the SHAP local explanation algorithm to detect importance of features for the PAD prediction in each country and for each year considered in the analysis.

The SHAP algorithm is based on the concept of the Shapley (SHAP) values, mutuated from cooperative game theory (Lundberg and Lee, 2017; Lundberg et al., 2020), and consists in a local model-agnostic post-hoc explainer, that learns local interpretable linear models for the samples, focusing on the contributions of each feature on the prediction of each sample. The SHAP value for a feature *j* is evaluated as the difference between the prediction of the model output with and without that specific feature, considering all possible feature subsets. Hence, the model must be retrained on all feature subsets *F* of the entire set *S* of features (*F* ⊆ *S*). If *f*_*x*_(*F*) represents the prediction *f* of a model for the instance *x*, given a subset *F* that does not include the *j*-th feature, and *f*_*x*_(*F*∪*j*) is the prediction of the same model when the *j*-th feature is added, the marginal contribution provided by the *j*-th feature can be computed as the difference *f*_*x*_(*F* ∪ *j*) − *f*_*x*_(*F*). The SHAP value of the *j*-th feature for the instance *x* is then assessed by considering the addition of the *j*-th feature to all possible subsets,

(5) $$\begin{array}{l} SHAP_{j}\left(x\right)=\underset{F\subseteq S-\left\{j\right\}}{\sum }\frac{|F|!\left(|S|-|F|-1\right)!}{|S|!}\left[f_{x}\left(F\cup j\right)-f_{x}\left(F\right)\right], \end{array}$$

where |*F*|! represents the number of permutations of features positioned before the *j*-th feature, (|*S*| − |*F*| − 1)! represents the number of permutations of feature values that appear after the *j*-th feature value and |*S*|! is the total number of feature permutations (Lundberg and Lee, 2017).

In this work, the SHAP values are referred to importance of the features selected by Boruta in the PAD prediction, made by the RF algorithm. In particular, we consider the mean SHAP values computed after a 5-fold cross validation, repeated 100 times for each year. Then, in view of the geographical analysis, we operate for each year a further selection, by

- Evaluating the average impact of each feature as the mean absolute SHAP value on all countries,
- Computing the total mean absolute SHAP value, by summing on all features,
- Retaining only the features whose mean absolute SHAP value is larger than 25% of the total.

## 3. Results

### 3.1. Boruta competition network

**Figure 3:**
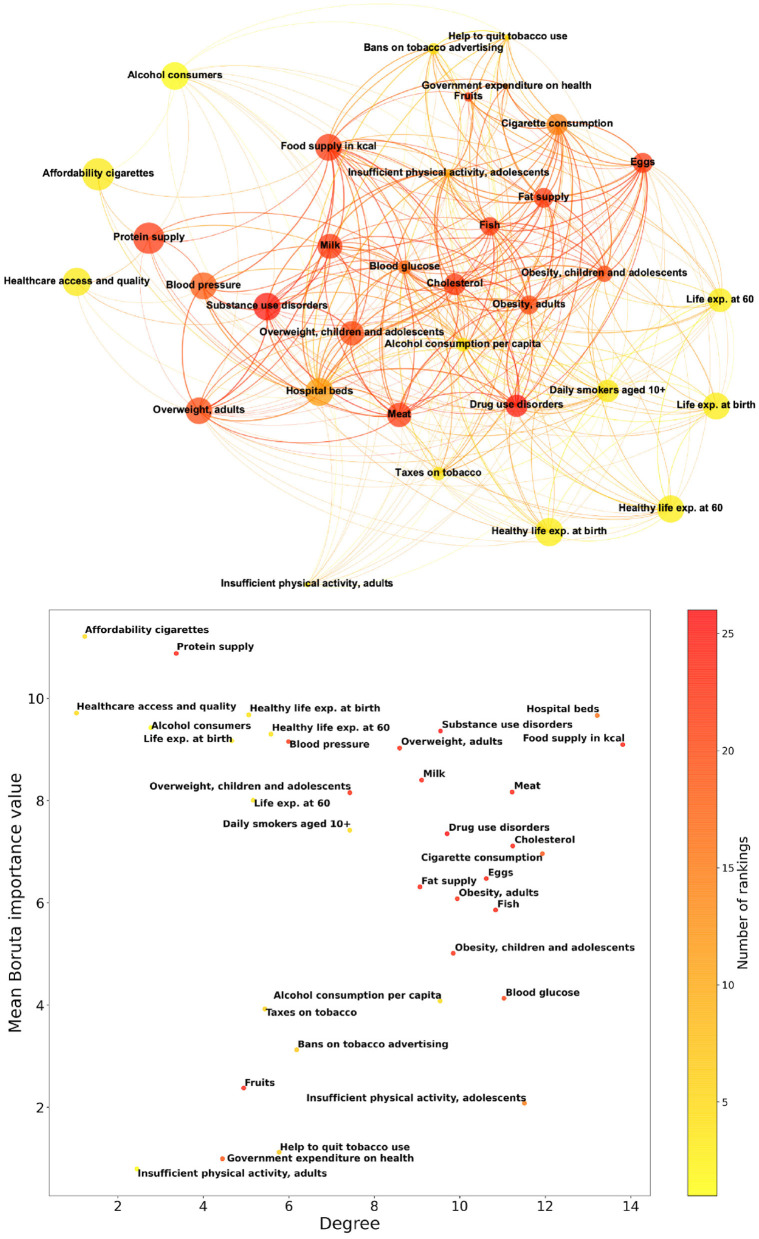
Properties of the competition network based on the rankings of Boruta feature importance values for different years. The upper panel shows the network structure, consisting of 34 nodes representing features, and 338 weighted edges that quantify the tendency of different features to switch their positions in the importance rankings. The lower panel reports in a scatter plot the network degree of features and their mean Boruta importance values. In both panels, features are colored according to the number of Boruta rankings in which they appear.

Starting from the Boruta feature importance, we construct a competition network, based on the rankings of importance values for different years. Figure 3 shows in the upper panel the competition network structure, consisting of 34 nodes representing features, and 338 weighted edges that quantify the tendency of different features to switch their positions in the Boruta importance rankings. Nodes shown in Figure 3 are characterized by colors that, in a scale from yellow to red, indicate the availability of the corresponding feature, and sizes that increase with the related Boruta feature importance values.

The lower panel of Figure 3 contains a scatter plot in which points correspond to features and their coordinates are competition network degree and mean Boruta importance value. Here, points organize according to a “horseshoe” pattern, in which features with low degree tend to have either high or low importance, while features with high degree tend to have intermediate importance. Also in this figure, the color scale corresponds to the number of yearly rankings in which a feature appears. The competition network analysis corroborates and integrates the results of Boruta importance: features that appear in the top left part of the scatter plot in Figure 3 and occur with high frequency in rankings, such as Protein supply, are steadily among the most relevant. On the other hand, frequent features appearing in the bottom left part, such as Government expenditure on health, can be safely considered irrelevant for dementia incidence prediction.

### 3.2. Random forest predictions

**Figure 4:**
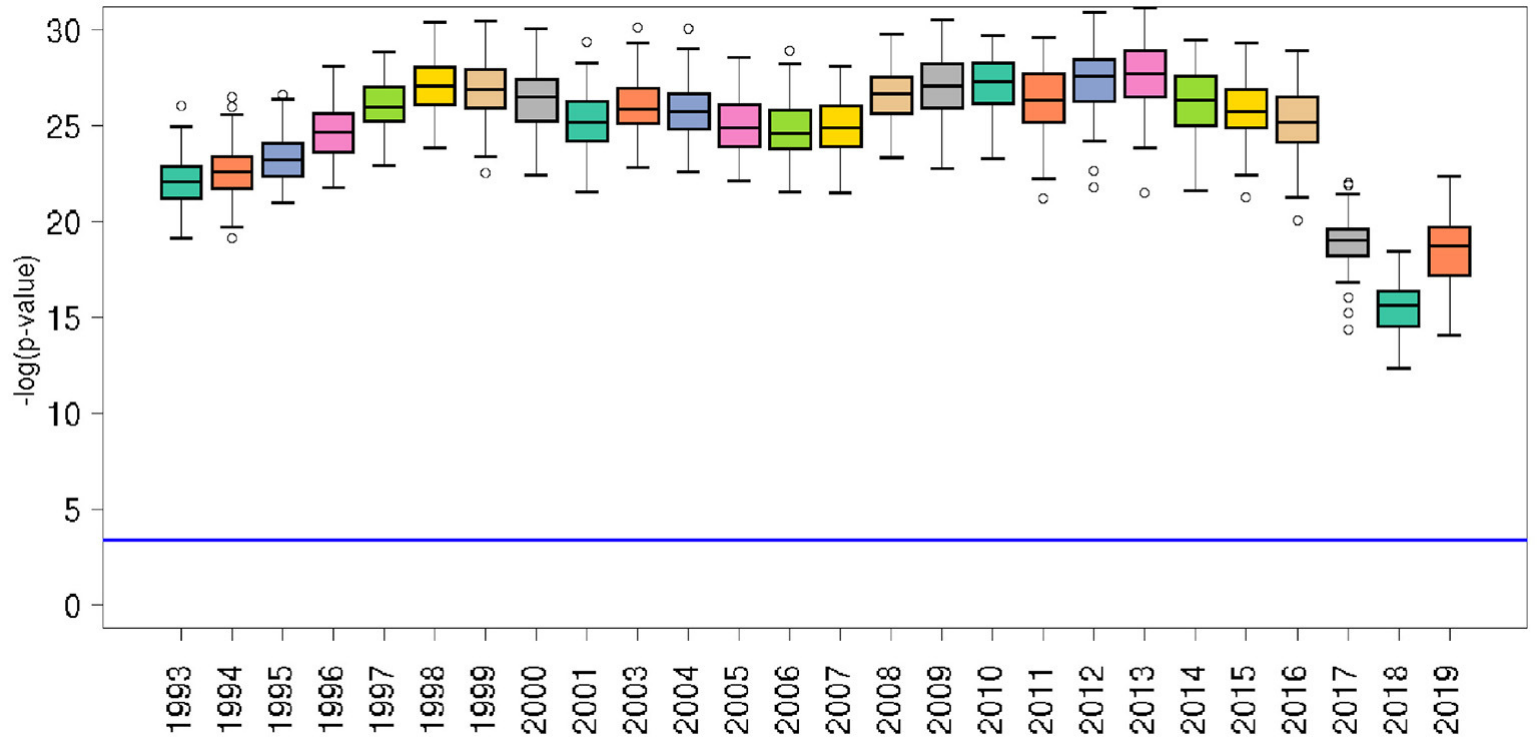
Significance of the RF model for each year, in terms of the relative *p*-value of the agreement (*R*^2^) between PAD actual values and RF predictions for each year. The horizontal blue line represents the *p* = 0.01 after correction for multiple hypothesis testing according to Bonferroni. Empty bullets represent distribution outliers.

We evaluate the effectiveness of predicting PAD through a RF regression algorithm, trained with the considered set of features. The performance of the regressor is quantified by the indicators described in Section 2.4. Figure 4 shows the relative *p*-value of the agreement (*R*^2^) between the PAD actual values and RF predictions for each year. The distributions reported in the plot are obtained by performing 100 cycles of 5-fold cross-validation, providing a total of 500 predictions. In the Supplementary material, we display in Supplementary Figures S1–S3 the related plots of *R*^2^, *RMSE*, and *MAPE*, respectively.

The behavior of all the performance indicators, reported in Table 1, shows that the predictive capability of the RF model is good, with fluctuations in time, until the year 2016, where a sudden increase of the root-mean-square error indicates a performance drop, followed in 2017 by all the other indicators. Such a change is due to the fact that the values of many relevant indicators are missing for the most recent years.

**Table 1:** Summary table of performance measures obtained for each year.

**Year**	***R* ^2^**	***MAPE***	***RMSE***
1993	0.503 ± 0.020	0.085 ± 0.002	66.075 ± 1.340
1994	0.513 ± 0.020	0.083 ± 0.002	65.465 ± 1.332
1995	0.525 ± 0.018	0.082 ± 0.002	64.731 ± 1.262
1996	0.541 ± 0.020	0.082 ± 0.002	63.867 ± 1.310
1997	0.562 ± 0.017	0.080 ± 0.002	62.831 ± 1.214
1998	0.574 ± 0.017	0.080 ± 0.002	62.605 ± 1.228
1999	0.570 ± 0.018	0.082 ± 0.002	63.385 ± 1.349
2000	0.566 ± 0.019	0.081 ± 0.002	64.029 ± 1.420
2001	0.552 ± 0.020	0.084 ± 0.002	65.073 ± 1.423
2003	0.563 ± 0.018	0.083 ± 0.002	64.186 ± 1.320
2004	0.560 ± 0.018	0.083 ± 0.002	64.349 ± 1.323
2005	0.548 ± 0.019	0.083 ± 0.002	65.230 ± 1.390
2006	0.543 ± 0.020	0.084 ± 0.002	65.516 ± 1.472
2007	0.547 ± 0.020	0.083 ± 0.002	65.240 ± 1.448
2008	0.569 ± 0.018	0.081 ± 0.002	63.702 ± 1.334
2009	0.572 ± 0.020	0.082 ± 0.002	63.575 ± 1.482
2010	0.573 ± 0.018	0.082 ± 0.002	63.610 ± 1.319
2011	0.561 ± 0.022	0.082 ± 0.002	64.631 ± 1.570
2012	0.573 ± 0.020	0.082 ± 0.002	63.987 ± 1.451
2013	0.580 ± 0.020	0.080 ± 0.002	63.709 ± 1.543
2014	0.561 ± 0.021	0.082 ± 0.002	65.509 ± 1.552
2015	0.554 ± 0.019	0.082 ± 0.002	66.288 ± 1.425
2016	0.545 ± 0.022	0.086 ± 0.002	67.242 ± 1.622
2017	0.450 ± 0.022	0.093 ± 0.002	74.172 ± 1.505
2018	0.383 ± 0.024	0.102 ± 0.002	78.142 ± 1.512
2019	0.442 ± 0.030	0.094 ± 0.002	73.510 ± 1.939

*R*^2^, *MAPE*, and *RMSE* were averaged over 100 rounds of cross-validation and reported with the respective standard deviations.

### 3.3. Feature importance

**Figure 5:**
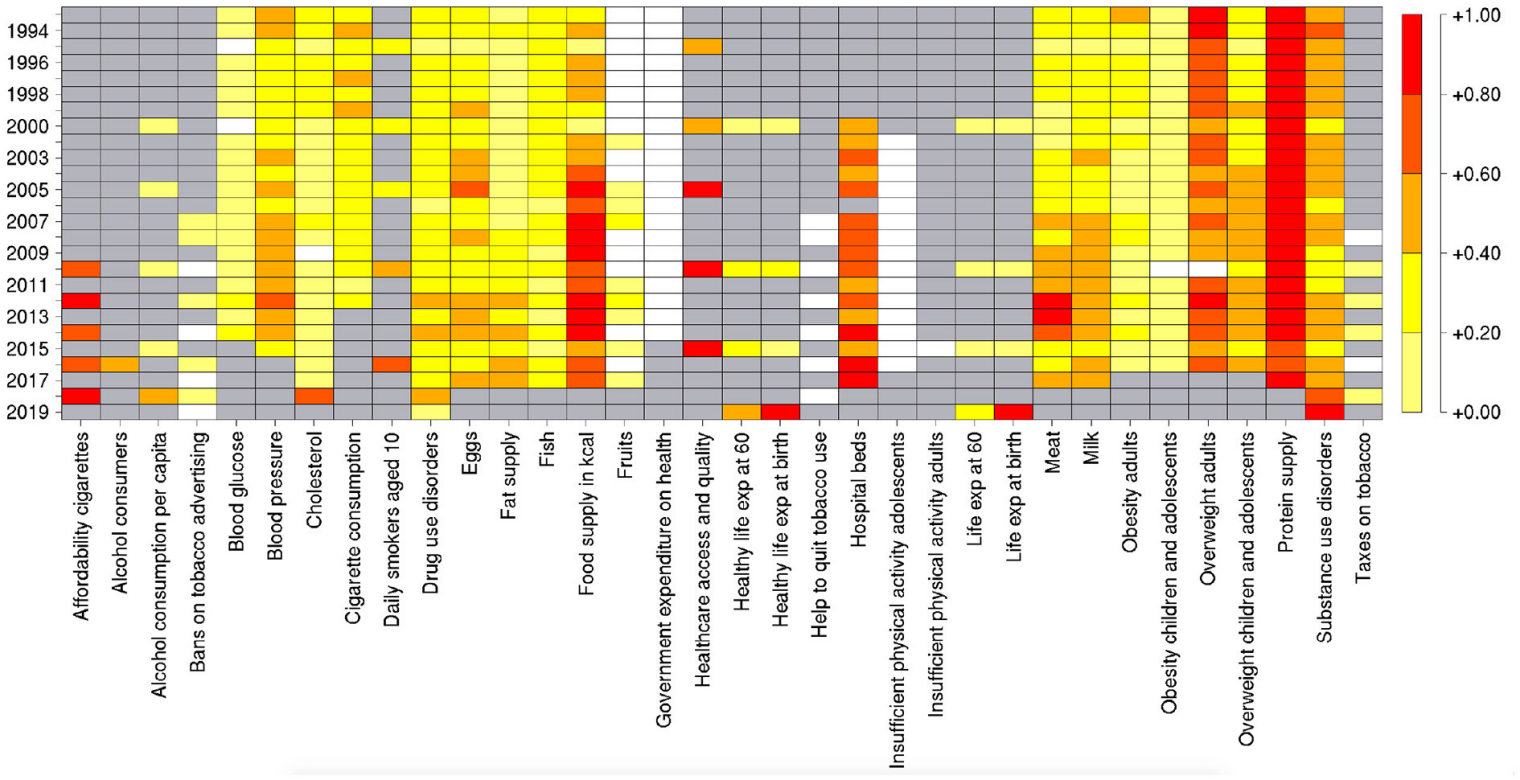
Average importance of the features used in the RF model over 100 repetitions of the 5-fold cross validation procedure, for 26 different years. The white boxes indicate that the corresponding feature was discarded in the preselection based on the Boruta algorithm. The gray boxes indicate that the corresponding indicators are missing for those years.

The results of the feature importance procedure performed through the RF algorithm are summarized in Figure 5, where the importance of features throughout the years is represented in a color scale from red (high) to yellow (low). White and gray boxes indicate that the corresponding features are either not selected by Boruta or missing for a given year, respectively. It is possible to detect that specific variables related to nutrition (Food supply in kcal, Overweight adults, Protein supply, and, especially for recent years, Meat) and to medical care (Hospital beds, Healthcare access and quality) tend to have a consistently high importance. In the last years, the number of unavailable indicators becomes larger, and RF tends to assign high importance to the remaining ones; however, as highlighted by the performance drop mentioned in Section 3.2, the predictive power of these indicators alone is low.

### 3.4. SHAP values

**Figure 6:**
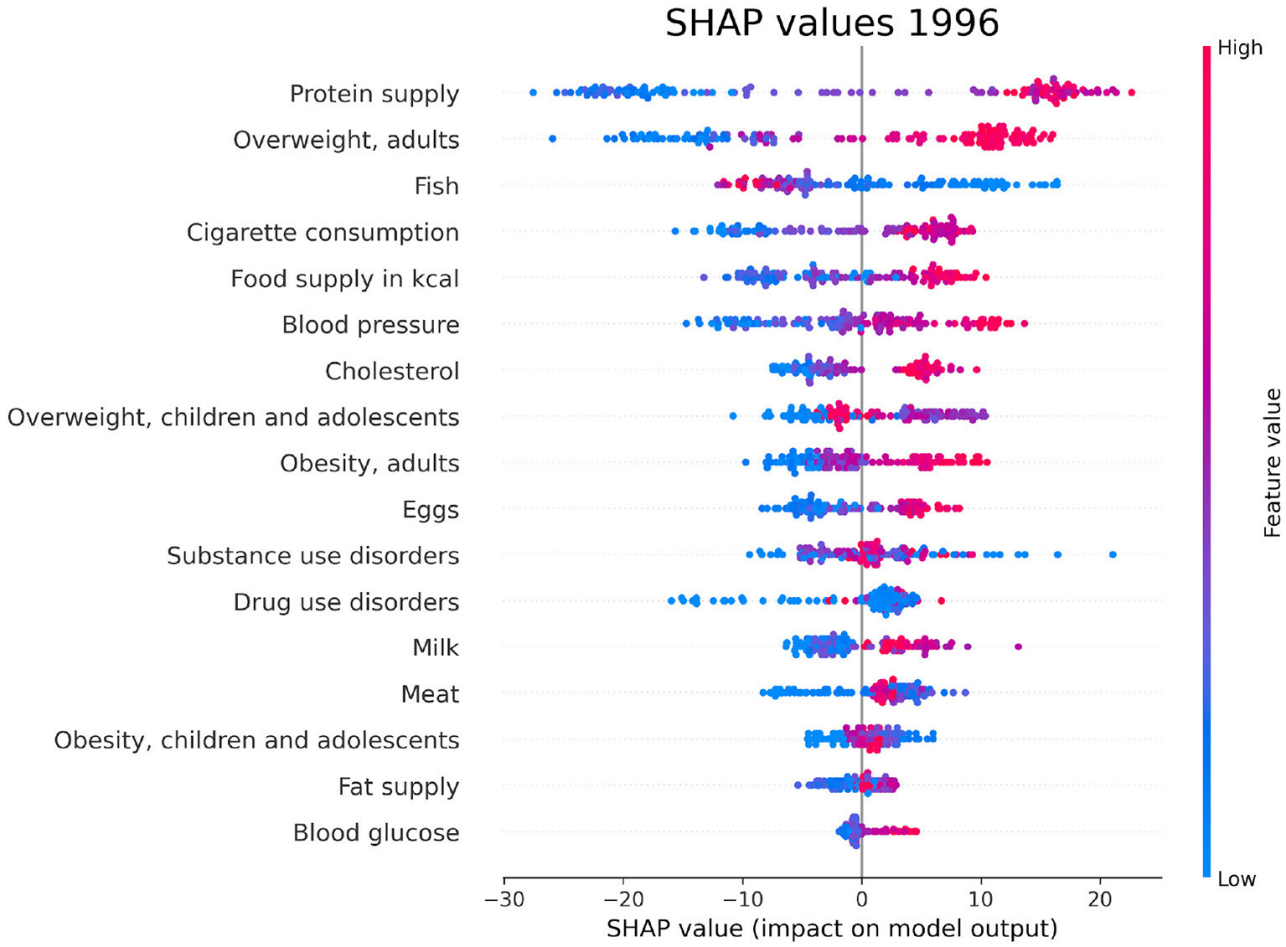
SHAP values corresponding to the features that are most influential in the prediction of PAD for the year 1996. Different points in the same row are associated to the prediction made for different countries.

**Figure 7:**
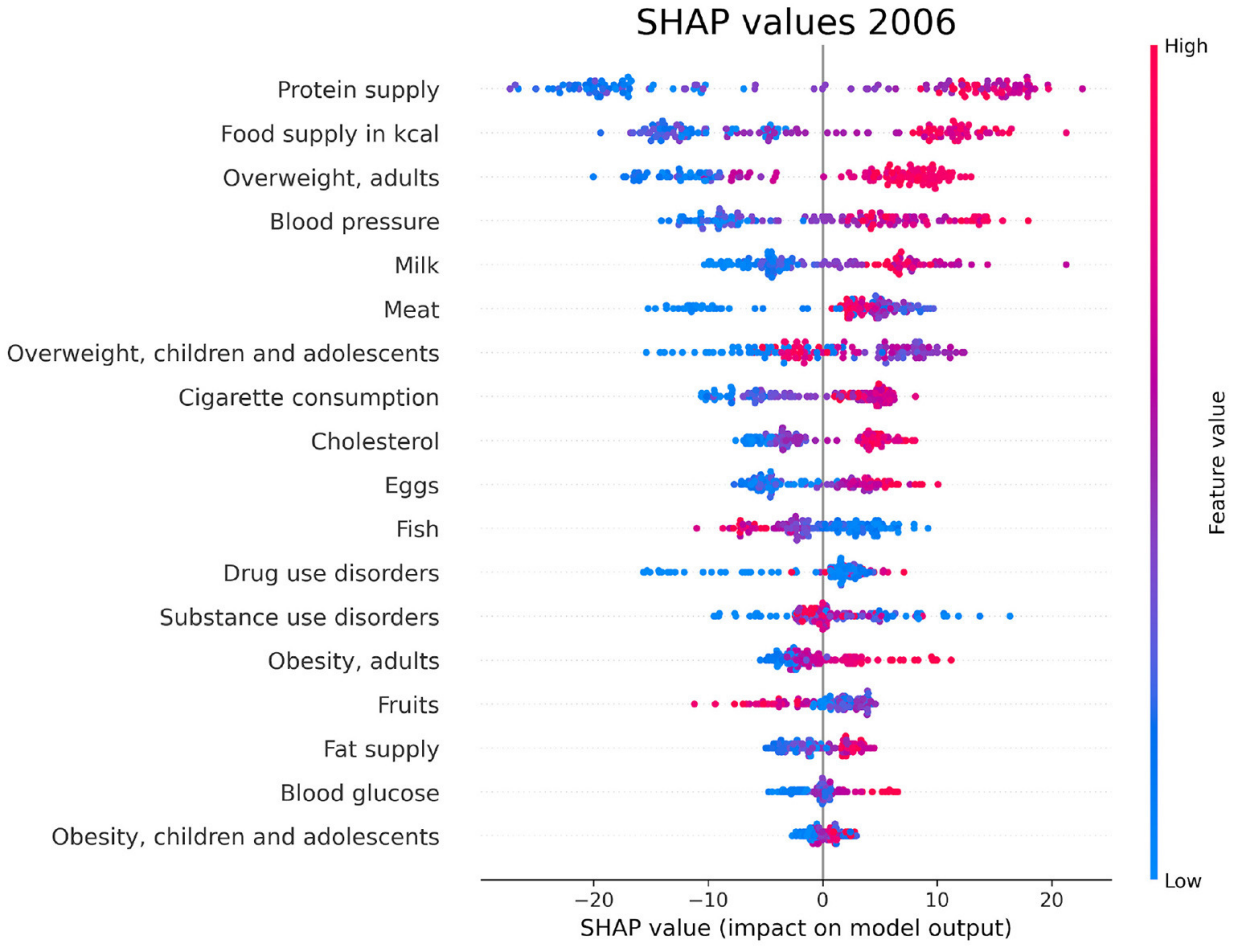
SHAP values corresponding to the features that are most influential in the prediction of PAD for the year 2006. Different points in the same row are associated to the prediction made for different countries.

**Figure 8:**
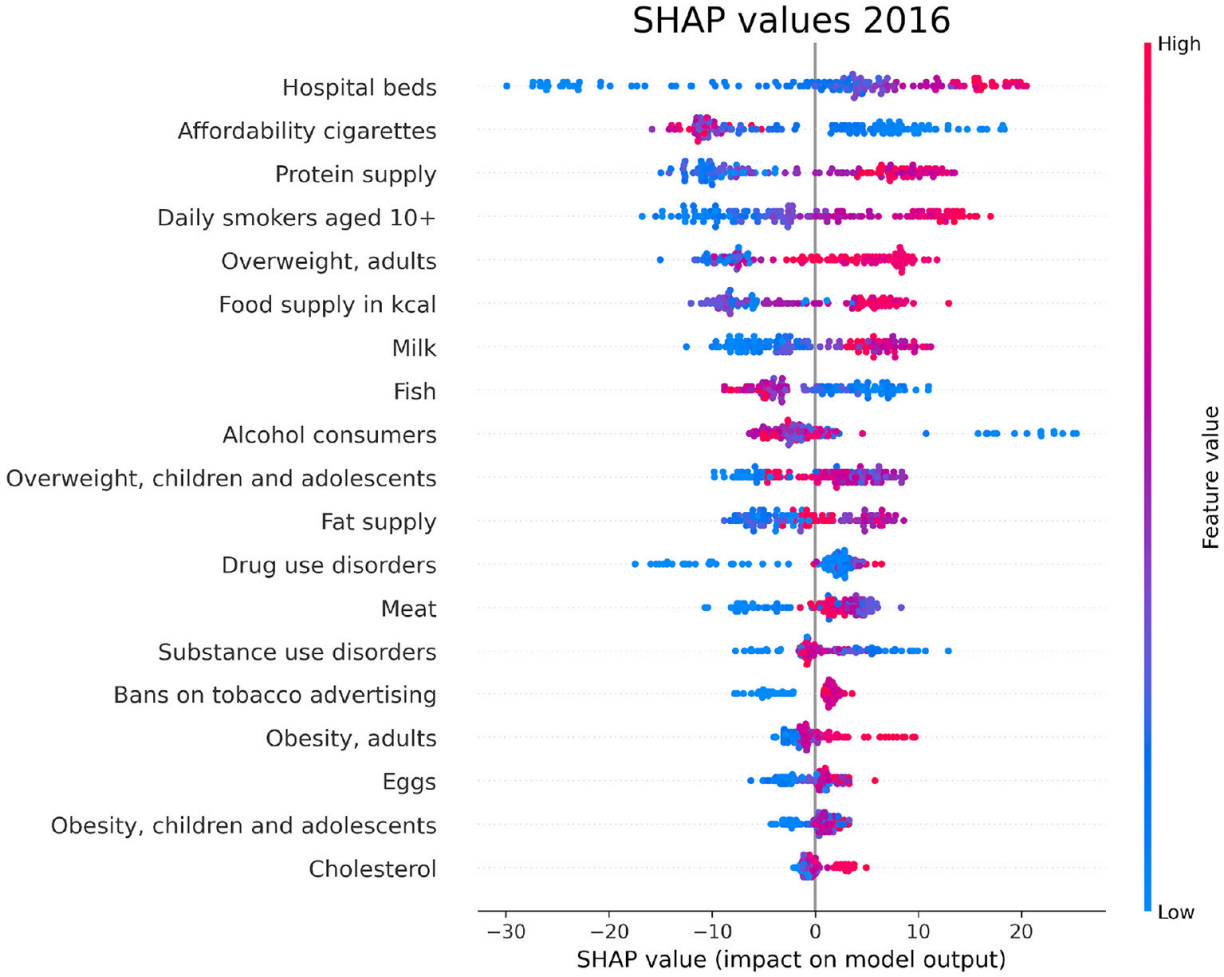
SHAP values corresponding to the features that are most influential in the prediction of PAD for the year 2016. Different points in the same row are associated to the prediction made for different countries.

For each year and each feature selected by Boruta, we compute the SHAP values associated to the PAD prediction for each given country. The plots in Figures 6–8 show the distribution of SHAP values for each selected feature, for the years 1996, 2006, and 2016. In these plots, the relevant features for the chosen year are ordered in terms of the mean absolute SHAP value, which indicates their overall impact on the dementia incidence prediction, independent of the way (positive or negative) in which they affect each outcome.

### 3.5. Geographical analysis

The SHAP values provide the basis for investigating the predictive power of the considered features from a geographical point of view, focused on single countries. As stated in Section 2.5, we retain only those features such that, in a given year, their mean absolute SHAP value is larger than 25% of the total absolute SHAP value. For the years 1993–2016, in which the RF model has a significant predictive power, this condition is satisfied in the following cases:

- Overweight, adults (1993–1999, and 2001);
- Protein supply (1993–2001, 2003–2009, 2011, 2013, and 2014);
- Food supply in kcal (2004, 2006–2009, 2011, 2013, and 2014);
- Meat (2013);
- Healthcare access and quality (2015);
- Hospital beds (2016).

**Figure 9:**
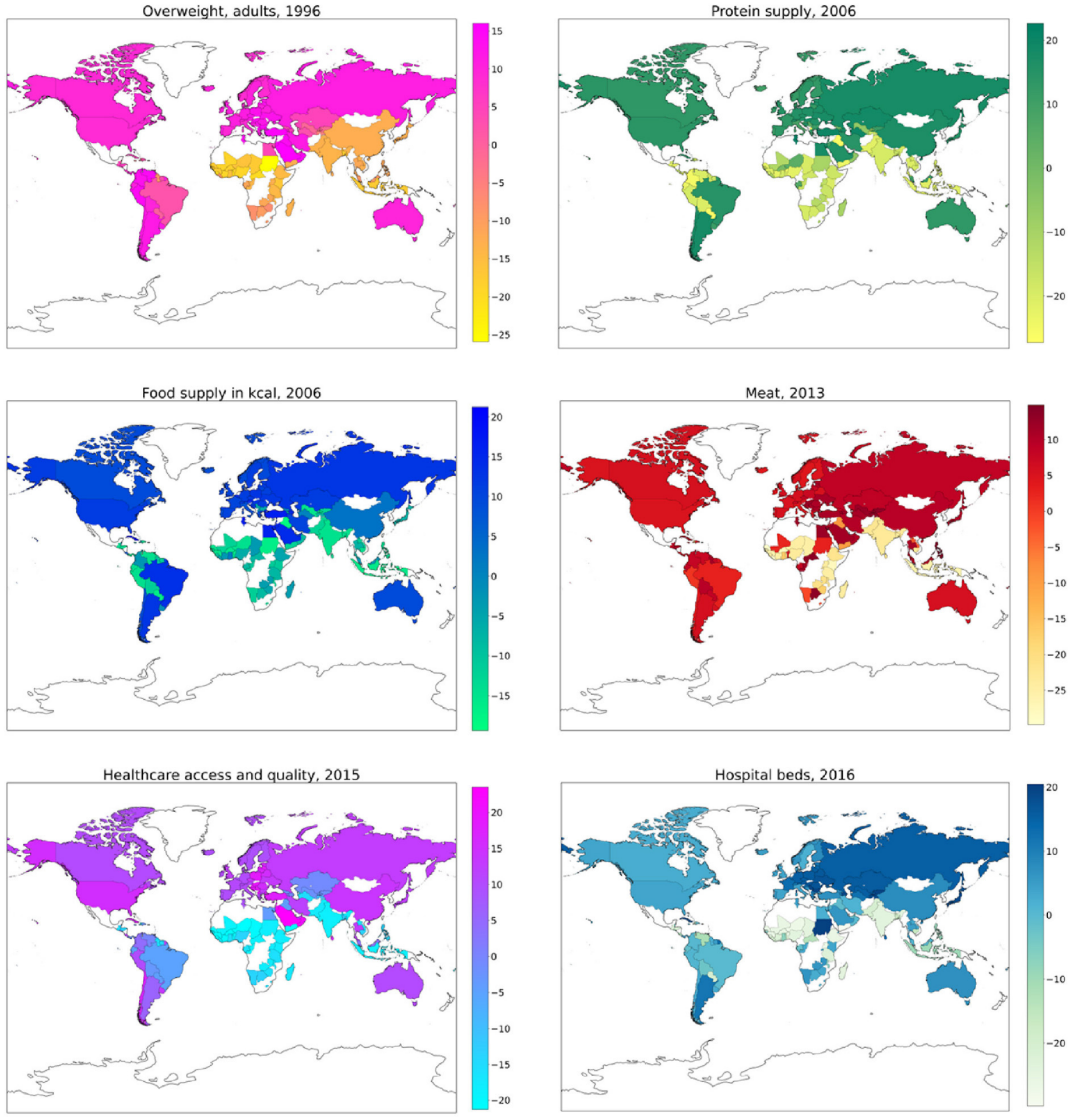
World maps of SHAP values associated with PAD prediction, related to the feature and year indicated in the each map label. Color bars are reported for numerical reference.

In Figure 9, we show the distribution in a world map of the SHAP values related to each of the above features, for one selected year. Since the results are qualitatively stable throughout the years, we preferably choose, when available, the values for one of the years 1996, 2006, 2016, to which Figures 6–8 are referred. The summary plots of SHAP values for the years 2013 and 2015, to which the maps related to Meat and Healthcare access and quality are referred, are reported in the Supplementary Figures S4, S5. We elaborate on the implications of these results in Section 4.

### 3.6. Inclusion of indicators not related to lifestyle

The analysis presented in this work is oriented to a comparison inside the set of lifestyle indicators as predictors of PAD. However, in order to test the robustness of its outcomes, we extend the range of indicators to check whether the lifestyle ones are still among the most influential to predict PAD. Specifically, we add to the dataset indicators in the following categories:

- Prevalence of confounding health disorders (“Depressive disorders,” “Autism spectrum disorders,” “ADHD,” “Type 2 Diabetes Mellitus”);
- Air pollution (“Fine particulate matter”);
- Schooling (“Average years of schooling,” “School life expectancy”);
- Country wealth (“GDP per capita”).

**Figure 10:**
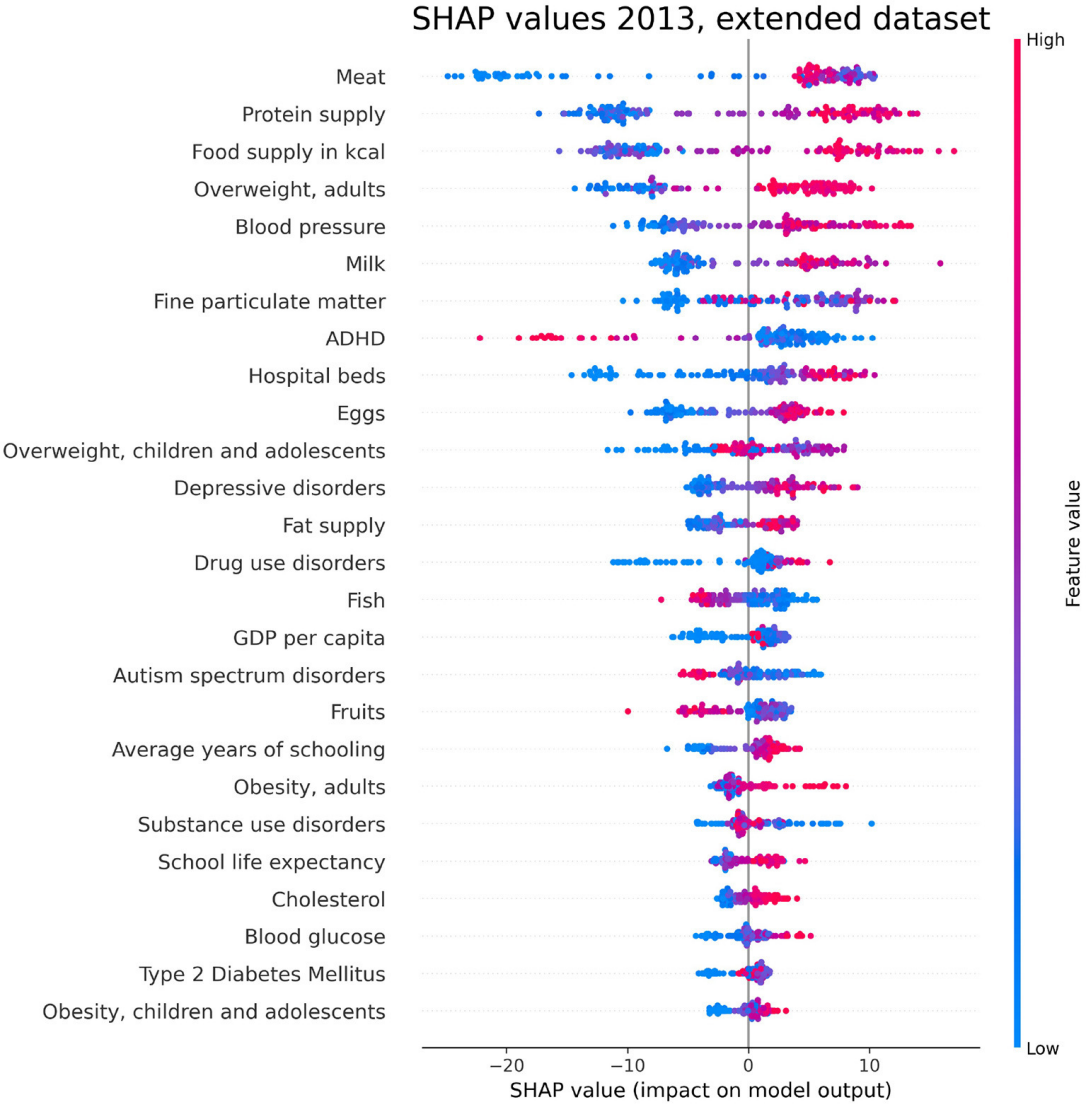
SHAP values corresponding to the features that are most influential in the prediction of PAD for the year 2013, obtained with the extended dataset described in Section 3.6. Different points in the same row are associated to the prediction made for different countries.

For indicator availability reasons, we test the extended dataset for the PAD regression related to the year 2013. Details on the sources of the additional indicators are reported in the Supplementary material. As expected, the RF performance slightly improves by extending the dataset, with *R*^2^ = 0.602 ± 0.020, *MAPE* = 0.078 ± 0.002 and *RMSE* = 62.071 ± 1.574 (see Table 1 for comparison). The top-ranked SHAP values for the extended dataset are reported in Figure 10, where it can be observed that lifestyle indicators are still among the most influential.

## 4. Discussion

We have investigated the factors that affect the prevalence of dementia in 137 UN countries, combining machine-learning based feature importance, the XAI approach, and the complex network formalism. First, we selected the most relevant variables by means of Boruta importance rankings, encompassing information concerning different data availability of the indicators through a competition network. From this part of the analysis, we found relevant features related to individual health parameters (overweight, blood pressure), life expectancy, which is trivially correlated with the onset of dementia, healthcare access and quality, substance use/abuse, and, most importantly, nutrition (protein supply, milk and meat consumption). On the other hand, among the least important features we found those related to government health expenditure, insufficient physical activity among adolescents, fruit consumption, and actions to discourage tobacco use. The importance of nutrition-related variables is emphasized also in the SHAP analysis, which highlights the impact of food supply in general, protein supply, and meat consumption, along with the percentage of overweight adults, on the prediction of dementia prevalence. From the plots in Figures 6–8, one can also notice that low values of fish consumption are associated with a higher incidence prediction.

The results of the SHAP values geographical analysis provide interesting insights when compared to the PAD world map in Figure 1. First, we must observe how the variables “Hospital beds” and “Healthcare access and quality” positively affect the prediction of PAD for countries in America, Europe, Australia, Central Asia, and some countries in Middle and Far East (including China and Japan). The obtained results indicate how the PAD can be underestimated in countries with a weak and poorly organized healthcare structure, where dementia diagnosis is more difficult. In the maps concerning overweight and nutrition, we can find interesting patterns that relate to specific features observed in Figure 1. We highlight in particular the following relevant cases:

- Higher SHAP values for the food supply and protein supply variables can explain the fact that Brazil, Argentina and Uruguay have larger PAD than surrounding countries in South America;
- The higher PAD for Thailand with respect to the surrounding countries is partly explained by meat consumption; however, one should also notice that a relevant role in the prediction for this country is played by the healthcare-related variables.
- The belt of African countries with high PAD in Figure 1, comprising Egypt, Sudan, Central African Republic, Republic of Congo, and Gabon, is characterized, as a whole, by high SHAP values for meat consumption.

The recurring relation between dementia incidence and dietary patterns finds many correspondences in literature, especially regarding AD. Previous works bring out a link between nutrition and both dementia and depression, together with the benefits of fruit consumption on the central nervous system (Moore et al., 2018). Moreover, a healthy diet such as the Mediterranean one is know as a protective factor against neurodegenerative diseases, and AD in particular (Gardener and Rainey-Smith, 2018; van den Brink et al., 2019), as it preserves neuronal synapses and delays cognitive impairment through the intake of nutrients with antioxidant, anti-inflammatory and free radical contrast properties (Dominguez and Barbagallo, 2016). On the other hand, processed meat consumption has been identified as a potential risk factor for dementia (Zhang et al., 2021), although there is no unanimous consensus on this result, since other studies relate very low meat consumption with a long-term risk of dementia and AD (Ngabirano et al., 2019). The central role of nutrition in the PAD prediction, emerging in our XAI framework, corroborates the necessity to implement prevention strategies based on the One Health approach (WHO, 2017b). This model proposes to take on the needs of the most vulnerable subjects, including people with disabilities, considering the deep relation between the well-being of individuals, animals and the ecosystem. The case of dementia prevention is emblematic in this regard, as it highlights connections between individual health and sustainability of food policies, especially concerning meat production and consumption.

The role of health parameters closely associated to nutrition, such as overweight and obesity, has already been recognized in previous literature. Specifically, a research by Singh-Manoux et al. (2018) showed that obesity at age 50 can increase the risk of dementia. This finding was corroborated by another study (Ma et al., 2020) associating increased body weight and abdominal obesity with increased dementia incidence. Another emerging factor in our analysis, namely tobacco use, has been investigated in studies which found that mortality associated with dementia is higher in smokers than in never smokers (Batty et al., 2014), and that the increased risk of dementia for smokers decreases after smoking cessation (Zhong et al., 2015). We finally observe that, as one can observe in Figure 10, the relevance of the considered lifestyle indicators in determining PAD prediction persists even after extending the dataset to the categories described in Section 3.6. Remarkably, the first occurrence of an additional variable, ranking seventh in terms of mean absolute SHAP value, is constituted by “Fine particulate matter,” which has been already identified as a predictor of dementia in previous literature (Peters et al., 2019).

## 5. Conclusions and outlook

In this research work, we focused on the prediction of PAD, an aggregated value representing prevalence of dementia in the population, available for a time period of 26 years, starting from a set of lifestyle indicators, referred to the same year of the prediction. As we previously observed, dementia starts developing with mild symptoms, and usually remains latent until their worsening. The delay between dementia onset and diagnosis depends on various factors, related to both the individual and the features of the healthcare system, such as diagnostic promptness and technological advance of medical instruments. Even the progression speed of the disease can be influenced by individual and environmental factors. Therefore, it is reasonable to think that dementia prevalence, quantified by PAD, takes into account people who became ill in different times, and are thus undergoing different stages of the disease. Moreover, lifestyle risk and protective factors can act on very different time scales, depending on their specific nature, e.g. associated with nutrition, on personal health parameters, or habits. Such an intrinsic variability in setting up the problem led us to the choice of training and testing the model for each fixed year. On the other hand, an extension perspective of this work concerns the possibility to predict PAD values referred to a specific advancement stage of the disease, using lifestyle indicators of previous years and optimizing the reference timescales.

The analysis performed in this work, based on the use of broad data in the aggregated form, can be extended with innovative information acquisition strategies, that can make the data collection procedure multimodal and more pervasive. A crucial contribution to mapping predictors of dementia onset can come from integrating deep data with routine health data collected by online patient platforms, but also from retailers and mobile phone providers, that allow to gain insights on individual social habits and lifestyle (Deetjen et al., 2015). This approach, already followed in other applications of Artificial Intelligence for Social Good to predict and monitor vulnerabilities related to poverty (Steele and et al., 2017), urban segregation (Lamanna et al., 2018), food insecurity (Martini et al., 2021), and gender inequalities (Garcia et al., 2018) represents a promising frontier to develop dementia prevention strategies, as well as decisional support and personalized medicine tools.

## Data availability statement

The original contributions presented in the study are included in the article/Supplementary material, further inquiries can be directed to the corresponding author.

## Author contributions

LB and AM: conceptualization, methodology, software, validation, investigation, data curation, writing–original draft preparation, and visualization. LB, AM, NA, AL, EP, ST, and RB: writing–review and editing. RB: supervision. All authors have read and agreed to the published version of the manuscript.

## Supplementary data

The Supplementary Material file reports a list of the indicators used for the analysis in the main text, with indication of their sources and their availability ranges, and the values of the additional performance indicators, that corroborate the evaluation of the PAD prediction made by the RF algorithm.
