# Supplementary material for: Worldwide impact of lifestyle predictors of dementia prevalence: An eXplainable Artificial Intelligence analysis
Source: Front Big Data. 2022 Dec 8;5:1027783. doi: 10.3389/fdata.2022.1027783 (PMC9772995; doi:10.3389/fdata.2022.1027783)
Supplement: Supplementary file 1 [file Data_Sheet_1.PDF]

Supplementary Material

1 INDICATOR DESCRIPTIONS AND SOURCES

We list below the indicators used for the analysis in the main text. Availability is reported starting from 1993; in some cases, ranges are wider than with those in Figure 7 of the main text, due to data selection processes. Data are retrieved from either the Global Health Observatory data repository of the World Health Organization (WHO, 2022) or the Our World in Data repository (Global Change Data Lab, 2022).

1. **Affordability of cigarettes**: percentage of GDP *per capita* required to purchase 2000 cigarettes of the most sold brand. Availability: 2010-2020. Data published by Global Health Observatory - World Health Organization (2022).
   Link: https://ourworldindata.org/grapher/affordability-cigarettes.
2. **Alcohol consumers**: share of adults who drank alcohol in last year. Availability: 2016. Data published by Global Health Observatory - World Health Organization (2022).
   Link: https://ourworldindata.org/grapher/share-of-adults-who-drank-alcohol-in-last-year
3. **Alcohol consumption per capita** (liters of pure alcohol, projected estimates, 15+ years of age). Availability: 2000, 2005, 2010, 2015, 2018. Data published by World Development Indicators - World Bank (2022).
   Link: https://ourworldindata.org/grapher/total-alcohol-consumption-per-capita-litres-of-pure-alcohol
4. **Bans on tobacco advertising**: assessment of country's legislation to determine whether all or any forms of tobacco advertising promotion and sponsorship are banned. Availability: 2007-2018.
   Link: https://ourworldindata.org/grapher/enforcement-of-bans-on-tobacco-advertising
5. **Blood glucose**: raised fasting blood glucose (≥ 7.0 mmol/L or on medication), age-standardized. Availability: 1993-2014. Data published by Global Health Observatory - World Health Organization (2022).
   Link: https://apps.who.int/gho/data/node.main.NCDRGLUCA?lang=en
6. **Blood pressure**: raised blood pressure (SBP≥ 140 or DBP≥ 90), age-standardized (%). Availability: 1993-2015. Data published by Global Health Observatory - World Health Organization (2022).
   Link: https://apps.who.int/gho/data/node.main.A875STANDARD?lang=en
7. **Cholesterol**: mean total cholesterol in mmol/l, averaged on subjects of 18+ age and both sexes, age standardized. Availability: 1993-2018. Data published by Global Health Observatory - World Health Organization (2022).
   Link: https://apps.who.int/gho/data/node.main.MEANTOTALCHOLESTEROL?lang=en
8. **Cigarette consumption**: average cigarette consumption per smoker per day (all ages and both sexes combined). Availability: 1993-2012. Data published by IHME, GHDx.
   Link: https://ourworldindata.org/grapher/consumption-per-smoker-per-day
9. **Daily smokers aged 10+**: Age-standardised prevalence of daily smoking in populations aged 10 and older (%). Availability: 1993-2016. Data published by: Global Burden of Disease Collaborative Network. Global Burden of Disease Study 2016 (GBD 2016) Health-related Sustainable Development Goals (SDG) Indicators 1990-2030. Seattle, United States: Institute for Health Metrics and Evaluation (IHME), 2017.
   Link: https://ourworldindata.org/grapher/prevalence-of-daily-smoking-sdgs

10. **Drug use disorders**: substance use disorders, both sexes, age-standardized (%). Data published by: Global Burden of Disease Collaborative Network. Global Burden of Disease Study 2019 (GBD 2019) Results. Seattle, United States: Institute for Health Metrics and Evaluation (IHME), 2021.
Link: https://ourworldindata.org/grapher/share-with-alcohol-or-drug-use-disorders
11. **Eggs**: average supply of eggs across the population, measured in kilograms per person per year. Availability: 1993-2017. Data published by United Nations Food and Agricultural Organization (2020).
Link: https://ourworldindata.org/grapher/per-capita-egg-consumption-kilograms-per-year
12. **Fat supply**: daily average supply of fat across the population, measured in grams per person per day. Availability: 1993-2017. Data published by United Nations Food and Agricultural Organization (2020).
Link: https://ourworldindata.org/food-supply#fat-supply
13. **Fish**: average supply of fish and seafood across the population, measured in kilograms per person per year. Availability: 1993-2017. Data published by United Nations Food and Agricultural Organization (2020).
Link: https://ourworldindata.org/grapher/fish-and-seafood-consumption-per-capita
14. **Food supply in kcal**: supply of kilocalories per person per day. Availability: 1993-2017. Data published by Our World in Data based on UN FAO & historical sources.
Link: https://ourworldindata.org/food-supply#caloric-supply-by-region
15. **Fruits**: average supply of fruit across the population, measured in kilograms per person per year. Availability: 1993-2018. Data published by United Nations Food and Agricultural Organization (2020).
Link: https://ourworldindata.org/grapher/fruit-consumption-per-capita
16. **Government expenditure on health**: current expenditure on health by general government and compulsory schemes (% of current expenditure on health). Availability: 1995-2012. Data published by Global Health Observatory - World Health Organization (2022).
Link: https://apps.who.int/gho/data/node.main.HS05?lang=en
17. **Healthcare access quality index**: HAQ Index (IHME (2017)). Availability: 1990, 1995, 2000, 2005, 2010, 2015. Data published by The Lancet in May 2017 in "Healthcare Access and Quality Index based on mortality from causes amenable to personal healthcare in 195 countries and territories, 1990–2015: a novel analysis from the Global Burden of Disease Study 2015".
Link: https://ourworldindata.org/grapher/healthcare-access-and-quality-index
18. **Healthy life expectancy at age 60**: average number of years in full health a person (usually at age 60) can expect to live based on current rates of ill-health and mortality. Availability: 2000, 2010, 2015, 2019. Data published by Global Health Observatory - World Health Organization (2022).
Link: https://apps.who.int/gho/data/node.main.688?lang=en
19. **Healthy life expectancy at birth**: average number of years that a person can expect to live in full health by taking into account years lived in less than full health due to disease and/or injury. Availability: 2000, 2010, 2015, 2019. Data published by Global Health Observatory - World Health Organization (2022).
Link: https://apps.who.int/gho/data/node.main.688?lang=en
20. **Help to quit tobacco use**: national support for the cessation of tobacco smoking. Availability: 2007-2018. Data published by Global Health Observatory - World Health Organization (2022).
Link: https://ourworldindata.org/grapher/support-to-help-to-quit-tobacco-use

21. **Hospital beds**: hospital beds per 10000 population. Availability: 2000-2017. Data published by Global Health Observatory - World Health Organization (2022).
Link: https://apps.who.int/gho/data/node.main.HS07?lang=en
22. **Insufficient physical activity, adolescents**: percent of school going adolescents not meeting WHO recommendations on Physical Activity for Health, i.e. doing less than 60 minutes of moderate- to vigorous-intensity physical activity daily. Availability: 2001-2016. Data published by Global Health Observatory - World Health Organization (2022).
Link: https://apps.who.int/gho/data/node.main.A893ADO?lang=en
23. **Insufficient physical activity, adults**: percent of adults not meeting WHO recommendations on Physical Activity for Health, i.e. doing less than 60 minutes of moderate- to vigorous-intensity physical activity daily. Availability: 2016. Data published by Global Health Observatory - World Health Organization (2022).
Link: https://apps.who.int/gho/data/node.main.A893?lang=en
24. **Life expectancy at age 60**: average number of years that a person of 60 years old could expect to live, if he or she were to pass through life exposed to the sex- and age-specific death rates prevailing at the time of his or her 60 years. Availability: 2000, 2010, 2015, 2019. Data published by Global Health Observatory - World Health Organization (2022).
Link: https://apps.who.int/gho/data/node.main.688?lang=en
25. **Life expectancy at birth**: average number of years that a newborn could expect to live, if he or she were to pass through life exposed to the sex- and age-specific death rates prevailing at the time of his or her birth. Availability: 2000, 2010, 2015, 2019. Data published by Global Health Observatory - World Health Organization (2022).
Link: https://apps.who.int/gho/data/node.main.688?lang=en
26. **Meat**: Daily meat consumption per person (g). Availability: 1993-2016. Data published by Food and Agriculture Organization of the United Nations (2020).
Link: https://ourworldindata.org/grapher/daily-meat-consumption-per-person
27. **Milk**: average per capita milk consumption, measured in kilograms per person per year. Availability: 1993-2017. Data published by Food and Agriculture Organization of the United Nations (2020).
Link: https://ourworldindata.org/grapher/per-capita-milk-consumption
28. **Obesity, adults**: percentage of defined population with a body mass index (BMI) of 30 kg/m^2 or higher, both sexes, age-standardized. Availability: 1993-2016. Data published by Global Health Observatory - World Health Organization (2022).
Link: https://apps.who.int/gho/data/node.main.A900A?lang=en
29. **Obesity, children and adolescents**: percentage of defined population with a body mass index (BMI) greater than 2 standard deviation above the median, according to the WHO references for school-age children and adolescents. Availability: 1993-2016. Data published by Global Health Observatory - World Health Organization (2022).
Link: https://apps.who.int/gho/data/node.main.BMIPLUS2C?lang=en
30. **Overweight, adults**: percentage of defined population with a body mass index (BMI) between 25 and 30 kg/m^2 or higher, both sexes, age standardized. Availability: 1993-2016. Data published by Global Health Observatory - World Health Organization (2022).
Link: https://apps.who.int/gho/data/node.main.A897A?lang=en

31. **Overweight, children and adolescents**: percentage of defined population with a body mass index (BMI) between 1 and 2 standard deviations above the median, according to the WHO references for school-age children and adolescents. Availability: 1993-2016. Data published by Global Health Observatory - World Health Organization (2022).
    Link: https://apps.who.int/gho/data/node.main.BMIPLUS1C?lang=en
32. **Protein supply**: daily average supply of protein across the population, measured in grams per person per day. Availability: 1993-2017. Data published by United Nations Food and Agricultural Organization (2020).
    Link: https://ourworldindata.org/food-supply#protein-supply
33. **Substance use disorders**: Prevalence of substance use disorders, both sexes, age-standardized (%). Availability: 1993-2019. Data published by Institute for Health Metrics and Evaluation, Global Burden of Disease (2019).
    Link: https://ourworldindata.org/illicit-drug-use#prevalence-of-drug-use-disorders
34. **Taxes on tobacco**: share of tobacco retail price that is tax. Availability: 2008-2018. Data published by Global Health Observatory - World Health Organization (2022).
    Link: https://ourworldindata.org/grapher/share-of-tobacco-retail-price-that-is-tax

The following indicators are contained in the extended dataset for the year 2013, used to test the outcomes of the research with respect to the inclusion of additional variables not related to lifestyle.

1. **Depressive disorders**: incidence of depressive disorders (percent). Data published by Global Burden of Disease Collaborative Network in Global Burden of Disease Study 2019 (GBD 2019) Results.
   Link: https://vizhub.healthdata.org/gbd-results
2. **Autism spectrum disorders**: incidence of autism spectrum disorders (percent). Data published by Global Burden of Disease Collaborative Network in Global Burden of Disease Study 2019 (GBD 2019) Results.
   Link: https://vizhub.healthdata.org/gbd-results
3. **Attention-deficit/hyperactivity disorder**: incidence of attention-deficit/hyperactivity disorders (percent). Data published by Global Burden of Disease Collaborative Network in Global Burden of Disease Study 2019 (GBD 2019) Results.
   Link: https://vizhub.healthdata.org/gbd-results
4. **Diabetes mellitus type 2**: incidence of diabetes mellitus type 2 (percent). Data published by Global Burden of Disease Collaborative Network in Global Burden of Disease Study 2019 (GBD 2019) Results.
   Link: https://vizhub.healthdata.org/gbd-results
5. **Fine particulate matter in cities**: annual mean levels of fine particulate matter (e.g. PM2.5 and PM10) in cities (population weighted). Data published by WHO in WHO Urban ambient air quality database (2022).
   Link: https://unstats.un.org/sdgs/metadata/files/Metadata-11-06-02.pdf
6. **Average years of schooling**: Mean years of total schooling across all education levels. Published by United Nations Development Programme in Human Development Report (2018 Statistical Update).
   Link: https://ourworldindata.org/global-education#years-of-schooling
7. **School life expectancy**: number of years of schooling expected at entrance age if prevailing patterns of age-specific enrollment rates persist. Published by United Nations Development Programme in Human

Development Report (2018 Statistical Update).
Link: https://ourworldindata.org/global-education#the-world-is-more-educated-than-ever-before

8. **GDP per capita**: gross domestic product divided by midyear population (current US dollars). Published by World Bank in national accounts data.
Link: https://data.worldbank.org/indicator/NY.GDP.PCAP.CD

2 REASSIGNED INDICATOR VALUES

The following indicator values, available in the years 1993-2006 for the country *Serbia and Montenegro*, are assigned to Serbia (Montenegro is not in the list of selected states):

- Fish
- Fruits
- Eggs
- Milk
- Alcohol consumption per capita
- Hospital beds
- Insufficient physical activity (adolescents)
- Fat supply
- Protein supply
- Meat

The following indicator values, available in the years 1993-2016 for the country *Sudan (former)*, comprising the current Sudan and South Sudan, are assigned to Sudan (South Sudan is not in the list of selected states):

- Overweight (children and adolescents)
- Overweight (adults)
- Obesity (children and adolescents)
- Obesity (adults)
- Blood glucose
- Blood pressure
- Alcohol consumption per capita
- Fat supply
- Protein supply
- Meat

3 PERFORMANCE INDICATORS OF THE RANDOM FOREST PREDICTION

In order to corroborate the information provided in the main text, we show the values of the following performance indicators, that evaluate the PAD prediction made by the Random Forest algorithm: R^2 (Figure S1), RMSE (Figure S3), and MAPE (Figure S2).

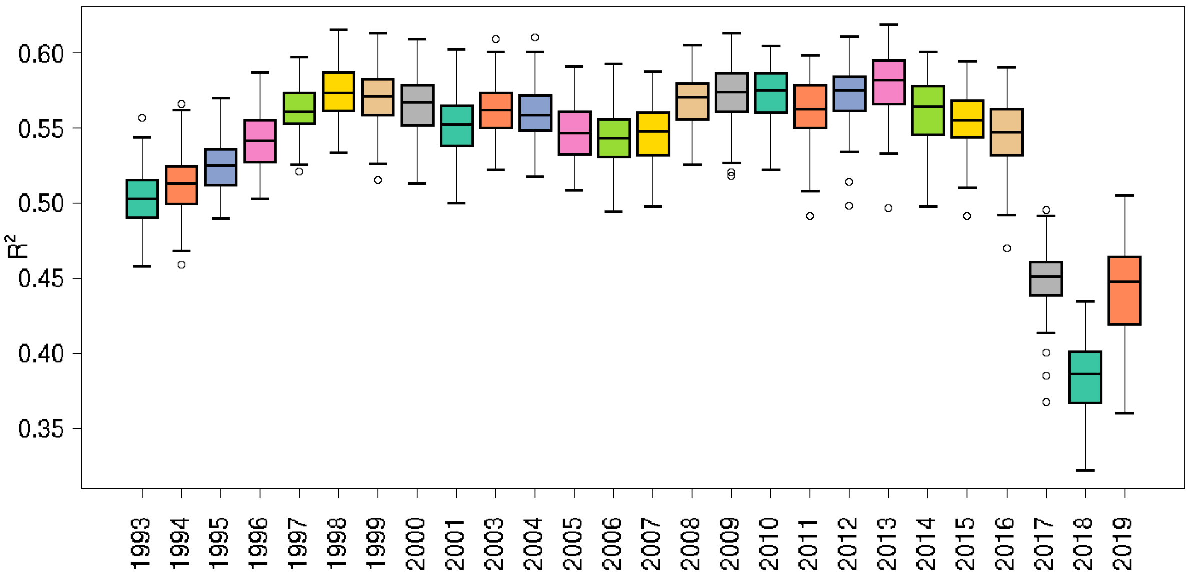

Figure S1. Boxplots of the agreement (R^2) between the PAD actual values and Random Forests prediction for each year. Distributions are obtained by means of a 5-fold cross validation procedure repeated 100 times. Empty bullets represent distribution outliers.

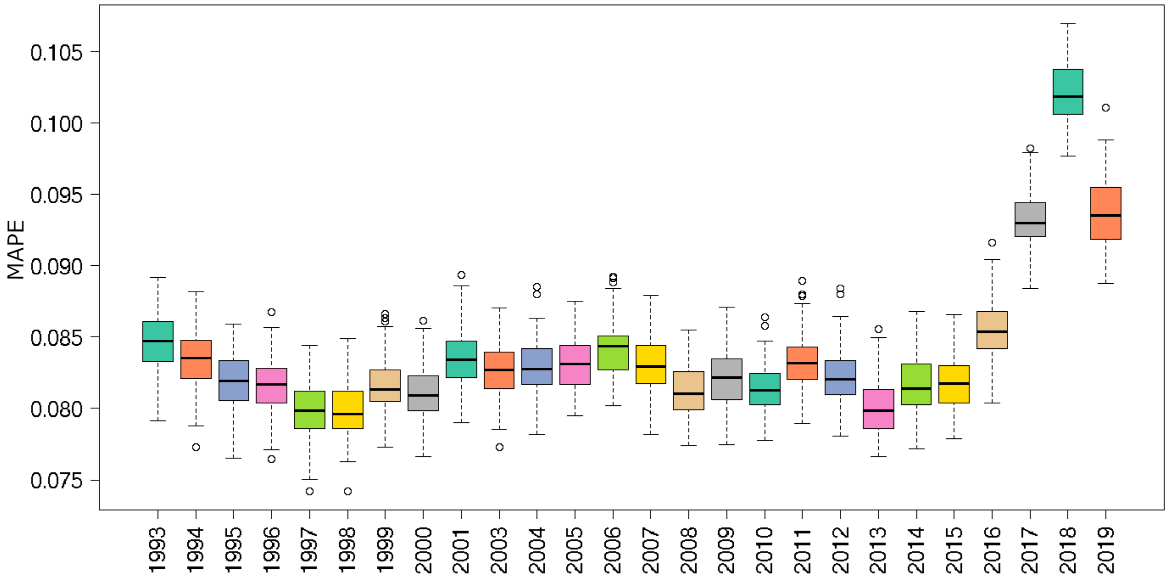

Figure S2. Boxplots of MAPE of the Random Forest model for each year. Distributions are obtained by means of a 5-fold cross validation procedure repeated 100 times. Empty bullets represent distribution outliers.

4 SHAP VALUES SUMMARY PLOTS FOR 2013 AND 2015

We show in Figures S4-S5 two complementary summary plots of SHAP values, reporting the distributions for the most relevant features related to the years 2013 and 2015, respectively.

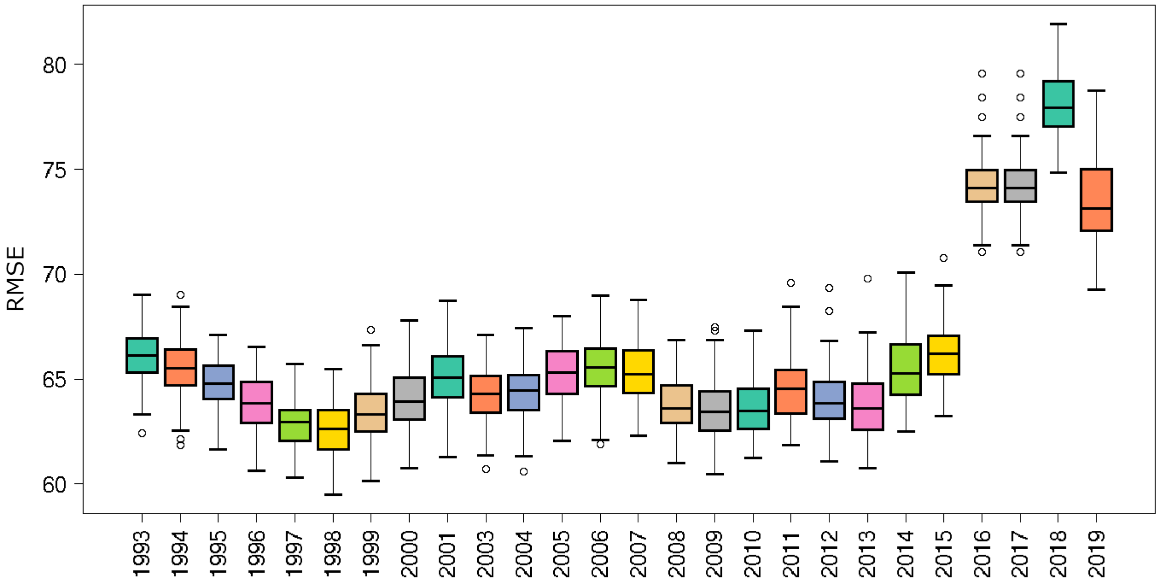

Figure S3. Boxplots of RMSE of the Random Forest model for each year. Distributions are obtained by means of a 5-fold cross validation procedure repeated 100 times. Empty bullets represent distribution outliers.

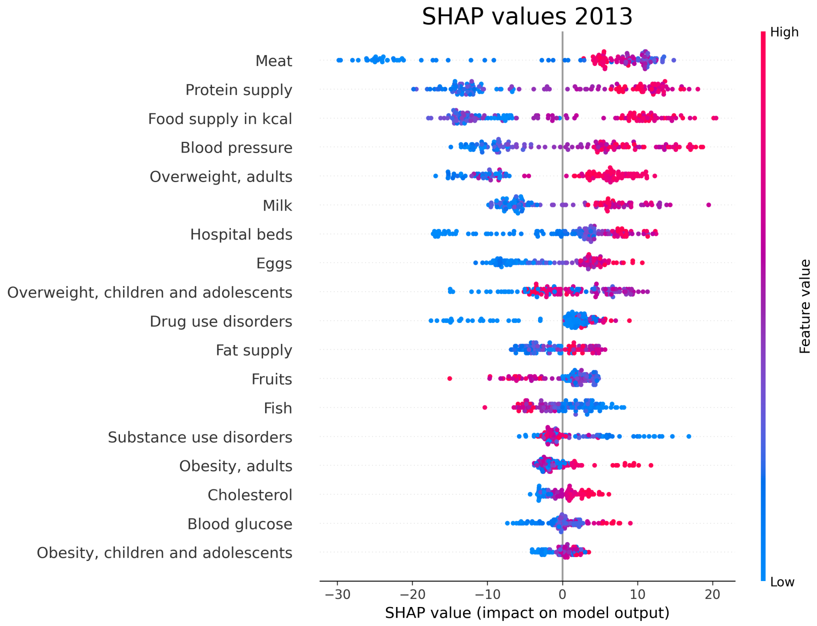

Figure S4. SHAP values corresponding to the features that are most influential in the prediction of PAD for the year 2013. Different points in the same row are associated to the prediction made for different counties.

REFERENCES

[Dataset] Global Change Data Lab (2022). Our World in Data. `https://ourworldindata.org`

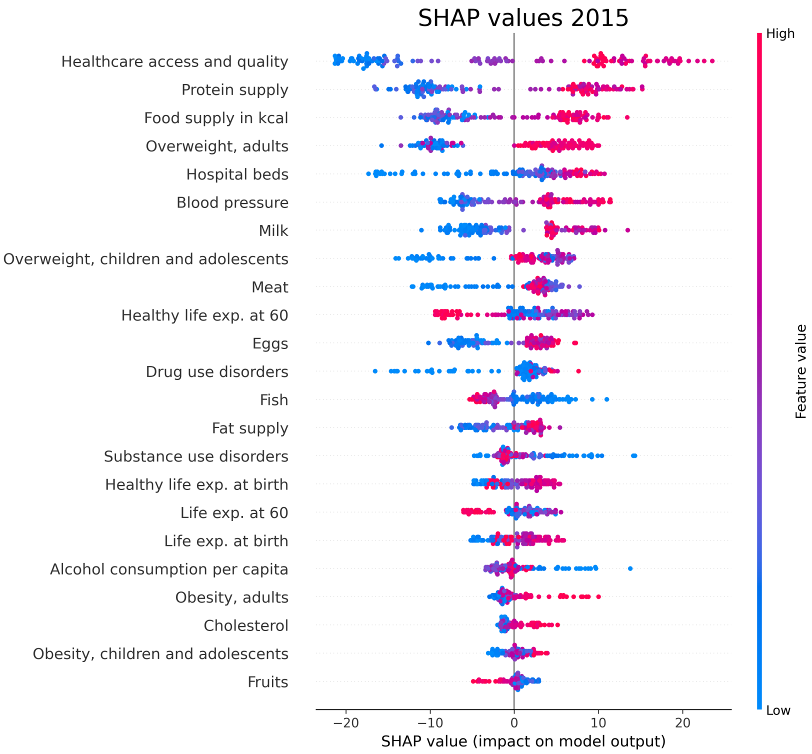

Figure S5. SHAP values corresponding to the features that are most influential in the prediction of PAD for the year 2015. Different points in the same row are associated to the prediction made for different counties.

[Dataset] WHO (2022). Global Health Observatory data repository. `https://apps.who.int/gho/data/node.home`
